# Development of highly efficient protocols for extraction and amplification of cytomegalovirus DNA from dried blood spots for detection and genotyping of polymorphic immunomodulatory genes

**DOI:** 10.1371/journal.pone.0222053

**Published:** 2019-09-12

**Authors:** Christian Berg, Martin B. Friis, Mette M. Rosenkilde, Thomas Benfield, Lene Nielsen, Hans R. Lüttichau, Thomas Sundelin

**Affiliations:** 1 Unit for Infectious Diseases, Department of Medicine, Herlev-Gentofte Hospital, University of Copenhagen, Herlev, Denmark; 2 Laboratory for Molecular Pharmacology, Department of Biomedical Sciences, Panum Institute, University of Copenhagen, Copenhagen, Denmark; 3 Department of Clinical Microbiology, Herlev-Gentofte Hospital, University of Copenhagen, Herlev, Denmark; 4 Department of Infectious Diseases, Hvidovre Hospital, University of Copenhagen, Hvidovre, Denmark; University of Helsinki, FINLAND

## Abstract

Congenital cytomegalovirus (CMV) infection is a major cause of birth defects ranging from developmental disorders to stillbirth. Most newborns affected by CMV do not present with symptoms at birth but are at risk of sequelae at later stages of their childhood. Stored dried blood spots (DBS) taken at birth can be used for retrospective diagnosis of hereditary diseases, but detection of pathogens is challenged by potentially low pathogen concentrations in the small blood volume available in a DBS. Here we test four different extraction methods for optimal recovery of CMV DNA from DBS at low to high CMV titers. The recovery efficiencies varied widely between the different extractions (from 3% to 100%) with the most efficient method extracting up to 113-fold more CMV DNA than the least efficient and 8-fold more than the reference protocol. Furthermore, we amplified four immunomodulatory CMV genes from the extracted DNA: the UL40 and UL111A genes which occur as functional knockouts in some circulating CMV strains, and the highly variable UL146 and US28 genes. The PCRs specifically amplified the CMV genes at all tested titers with sufficient quality for sequencing and genotyping. In summary, we here report an extraction method for optimal recovery of CMV DNA from DBSs that can be used for both detection of CMV and for genotyping of polymorphic CMV genes in congenital CMV infection.

## Introduction

Cytomegalovirus (CMV) is a widespread human herpesvirus that establishes lifelong latent infection and is a major cause of birth defects as well as morbidity and mortality in immunocompromised individuals [1]. In people living with HIV and organ transplant recipients, CMV infection may cause a multitude of debilitating and life-threatening organ diseases, most notably retinitis, colitis, and pneumonitis. During pregnancy, primary infection or reactivation of latent CMV reservoirs can lead to mother-to-child transmission, which can result in congenital CMV syndrome, characterized by a variety of symptoms including sensorineural hearing loss, vision impairment, mental retardation and stillbirth. Furthermore, congenital CMV syndrome is a potentially underdiagnosed disease with an estimated 87% of newborns with CMV infection being born asymptomatic [2]. These children are at risk of developing permanent sequelae at later stages of their childhood which occurs in as many as 13.5% of the cases [2]. The health burden of congenital CMV disease is considerable and has been estimated to $4 billion annually in the US by a National Academy of Science approved and National Institute of Health funded committee, ranking it in the most cost-effective group for vaccine development [3].

The CMV genome consists of monopartite, linear, double-stranded DNA and is roughly 235 kb in size. It contains more than 750 translated ORFs [4] which can be divided into two regions—the unique long (UL) and unique short (US) regions—flanked by terminal and internal inverted repeats. Cytomegalovirus has adapted a wide range of strategies to avoid immune detection and facilitate dissemination of infection. These strategies are based on manipulation and modulation of the host’s immune response during infection, e.g. by expression of virally encoded homologs of receptors and ligands important for the normal function of the human immune system. By encoding a 2 to 3-fold greater number of gene products than other human herpesviruses, many of which have been shown to interact with and manipulate the human immune system [5], CMV has an unparalleled number of tools available for modifying the host’s immune response. On the other hand, it could be an evolutionary disadvantage to have and maintain such a large genome. The genetic variation between circulating CMV strains is large and a recent study reported that 75% of the strains contain disruptive mutations and polymorphisms in several genes [6]. In order to exclude disruptive mutations due serial passage, the authors of the study only used strains passaged 1–2 times and verified most of the found mutations directly from clinical samples. For the genes UL40 and UL111A, mutations causing functional knockouts were found in 9.9% and 5.5% of the investigated strains, respectively [6]. UL111A is a functional interleukin-10 homolog that can inhibit a normal immune response [5, 7]. The signal peptide of UL40 facilitates surface expression of HLA-E on infected cells, which is a ligand for a natural killer cell inhibitory receptor [8]. Other CMV genes are highly variable such as the chemokine homolog UL146 where 14 distinct genotypes have been identified [9], and the chemokine scavenging receptor [10] and novel drug target US28 [11] where numerous N-terminal polymorphisms have been reported [12, 13]. This degree of genetic diversity is not observed for other human herpesviruses [6] and poses the question of why CMV exerts such variability among important immunomodulatory genes and how it affects the virus-host interaction. Genotyping of immunomodulatory CMV genes can serve as a tool for investigating this phenomenon by evaluating the virulence and pathogenicity of clinical strains that have adapted different genotypes of these immunomodulators.

In many countries, a blood sample is taken from newborns (neonatal heel prick) and stored on filter paper as dried blood spots (DBS) for screening of selected congenital diseases (also known as “Guthrie cards”). In Denmark, these cards are stored in a national biobank and can be accessed for both diagnostic and research purposes [14]. A number of research groups have previously tried to establish whether DBSs can be used to diagnose congenital CMV using different extraction and amplification protocols (S1 Table), but the reported sensitivities show considerable variation [15–29]. Using the DBS approach for detection of CMV poses a key problem in the very limited sample volume of which only a small part can be acquired due to the finite nature of these historically important samples. Thus, investigating the significance of the CMV’s genetic diversity in congenital disease by genotyping from DBSs requires a highly efficient extraction protocol and sensitive PCRs for the genes of interest. Such a setup has, to our knowledge, not previously been reported for DBSs [13, 30–42]. Here, we report a highly efficient method for extraction of CMV DNA from DBSs that is useful for detection of CMV in a limited sample volume. Furthermore, we show that this method can be used together with sensitive PCR procedures designed to identify functional knockouts and polymorphisms of the four CMV genes UL40, UL111A, UL146, and US28.

## Materials and methods

### Clinical samples

All clinical samples used in this study were sent for routine investigation to the Department of Clinical Microbiology (Herlev-Gentofte Hospital, Denmark) and afterwards stored for use in quality control and method development and optimization at the department. The study is approved by the Danish National Committee on Health Research Ethics (journal no. H-15017153). All samples were fully anonymized and only virus DNA was analyzed. The following CMV positive samples were included: one saliva sample, three serum samples, three bronchoalveolar lavage fluid samples, one amniotic fluid sample and 16 urine samples.

### Simulated Guthrie cards

A drop of fresh capillary blood from a healthy, voluntary, adult donor was placed on a piece of Parafilm M (Pechiney Plastic Packaging, Menasha, USA). For the 1:10 dilution, a CMV positive amniotic fluid sample with a known virus concentration (approximately 100,000,000 cp/ml by qPCR as described below) was diluted 1:10 in whole blood. For the 1:100 and 1:1000 dilutions, the CMV positive amniotic fluid stock was first diluted 10- and 100-fold (stepwise) in PCR grade water, which were then diluted 10-fold in whole blood (giving 1:100 and 1:1000 dilutions). From the resulting dilutions, 70 μl was spotted on Ahlstrom grade 226 filter paper cards, left to dry for approximately 3 h at RT, and the cards were hereafter stored at -20°C until analysis. From each dried blood spot, ten circular 3.2 mm diameter punches were made. DNA was extracted from either one or two of these punches (as specified below).

### Extractions

Extraction of DNA from the simulated Guthrie cards was evaluated using four different extraction protocols. Each protocol was evaluated using both one and two 3.2 mm punches.

Method A: Filter paper punches were incubated in 1,000 μl easyMAG lysis buffer (bioMérieux, Marcy-I’Étoile, France) for 10 min at RT with vortexing every 2 min. DNA was afterwards extracted from the lysate using a NucliSens easyMAG (bioMérieux) according to manufacturer guidelines, using the Specific A protocol without the initial internal lysis step. In brief, this protocol includes five washing steps and a DNA elution step with incubation in elution buffer for 10 min at 70°C. 100 μl NucliSens easyMAG Magnetic Silica (bioMérieux) was added to each extraction vessel and an elution volume of 70 μl was chosen.

Method B: Filter paper punches were incubated in 50 μl PBS+BSA (0.04%) overnight at 4°C. The next day, 150 μl PCR-grade water was added to the sample and DNA was extracted using the NUCLISENS easyMAG (bioMérieux) and the Specific A protocol as described for method A but including the lysis step.

Method C: Filter paper punches were placed in a PowerBeat tube (Qiagen, Hilden, Germany) with 0.1 mm glass beads. Then, 1,000 μl easyMAG lysis buffer (bioMérieux) was added and the sample was mixed on a Multi Reax shaker (Heidolph Instruments, Schwabach, Germany) for 5 min with the tube facing up and 5 min inverted. Approximately 900 μl lysate was transferred to a NUCLISENS easyMAG (bioMérieux) and the DNA was extracted using the NUCLISENS easyMAG (bioMérieux) and the Specific A protocol as described for method A.

Method D: Filter paper punches were incubated in 1,000 μl lysis buffer (600 μl PBS BSA (0.04%), 360μl Bacterial Lysis Buffer (Roche, Mannheim, Germany) and 40 μl Proteinase K (Roche) for 1 h at 55°C with vortexing every 2 min during incubation. DNA was afterwards extracted from the lysate using the NUCLISENS easyMAG (bioMérieux) and the Specific A protocol as described for method A with an additional internal lysis step.

### Quantitative PCR (qPCR)

CMV viral loads (cp/ml) of the extracted samples were quantified with the Argene CMV R-gene kit (bioMérieux) according to manufacturer’s guidelines using a Rotor-gene Q (QIAGEN) real-time PCR cycler.

### Primer design and optimization of PCR conditions

**Fig 1:**
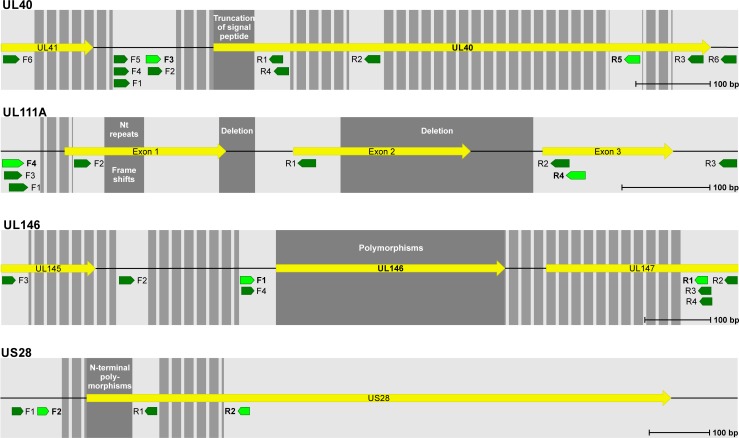
Overview of target genes and placement of all primers tested. Yellow arrows symbolize CMV genes and green arrows primers tested (F = forward, R = reverse). Striped regions were not suitable for primer placement due to nucleotide repeats, GC content, or inter-strain variations. Dark grey regions are the target areas for amplification. The best performing primers are marked in light green color and bold text. All elements are in a 1:1 scale.

For each CMV gene (UL146, UL111A, UL40 and US28), primers were manually designed (Fig 1 and S2 Table) and hereafter checked for homology with the human genome and the genome of CMV using BLAST [43]. All primers were ordered from TIB molbiol (Berlin, Germany) and several primer combinations were evaluated for their pairwise performance in PCR. To determine the optimal PCR conditions, the primer combinations were tested with varying annealing temperatures (in a ±5°C range of the theoretical T_m_) and with varying number of cycles (25–40 cycles). As a part of the optimization process, the following DNA polymerases were tested: Pfu DNA polymerase (Promega, Madison, USA), Herculase II Fusion Enzyme (Agilent Technologies, West Cedar Creek, USA), AmpliTaq Gold (Applied Biosystems, Branchburg, USA) and Phusion (Thermo Scientific, Waltham, USA).

### PCR amplification and analysis

The described PCR optimization resulted in the following conditions that were found to give the best results: Each PCR reaction was carried out in a total volume of 50 μl using 5 μl extracted DNA. For UL146, UL111A, and US28, the PCR mixture contained 1X *Pfu* DNA polymerase buffer with 2 mM MgSO_4_, 0.2 mM dNTP (each, Qiagen, Hilden, Germany), 0.5 μM of both forward and reverse primer (primer sequences can be seen in Table 1), and 1.25 U *Pfu* polymerase. For UL40, the PCR reaction mixture contained 1X Herculase II Reaction Buffer, 0.25 mM dNTP (each, supplied with the polymerase), 0.25 μM of both forward and reverse primer, and 0.5 μl Herculase II Fusion DNA Polymerase. Handling of PCR reagents and preparation of master mixes was carried out in a flow bench in a dedicated pre-PCR room.

**Table 1:** Primers used for amplification of CMV target genes.

Primer target	Forward sequence (5’-3’)	Reverse sequence (5’-3’)	Primer size (bp)	Annealing temp. (°C)	Elongation time (min)	Amplicon size (bp)
**UL40**	CTCTGTCTCGTCGTCATTC	GAATGCCCACAGTGTACATG	19 / 20	57	1.5	661
**UL111A**	CATCATAACATAAAGGACCACCTAC	CTGAGACAGCCGACTAATCAC	25 / 21	61	1.5	443–662
**UL146**	CCGGGAATACCGGATATTACG^1^	CAGCACTTCCTGACGATTG^1^	21 / 19	61	2	722–936
**US28**	CCGCTCATATAGACCAAACC	AGGGAGTTGTGATCTAGGAG	20 / 20	60	1	387

Sequences, PCR conditions, and expected amplicon sizes for primers used in the amplification of specified CMV genes.

^1^Primers previously reported by Dolan *et al*. [9].

The PCR amplification was performed using a ProFlex PCR System (Applied Biosystems, Foster City, USA) thermo cycler. An initial denaturation at 95°C for 2 min was followed by 40 cycles of denaturation at 95°C for 30 s, annealing at 57–61°C for 30 s, and elongation at 72°C for 1–2 min (see Table 1 for specific times and temperatures for each reaction); and finally 5 min of final elongation at 72°C. Samples were stored at 4°C until analyzed by capillary electrophoresis.

After amplification, 10 μl of the PCR product was analyzed on a QIAxcel Advanced System (Qiagen) capillary electrophoresis system using the AM420 screening method, 15 sec injection time and one run per row. The QIAxcel DNA Screening kit (Qiagen) was used in combination with the QX Alignment marker 50 bp/5kb (Qiagen) and QX Size marker 100 bp–2.5kb (Qiagen). Afterwards, the results were analyzed with the QIAxcel ScreenGel 1.6 software.

Selected positive samples were purified using the MinElute PCR Purification kit (Qiagen) according to manufacturer instructions before they were Sanger-sequenced by GATC Biotech (Köln, Germany) using the same primers as for PCR amplification (both forward and reverse). Sequences were aligned and analyzed using the Geneious R10 software.

### Calculations and graphics

The GraphPad Prism 7 software was used to calculate row means and standard errors of the mean (SEM), curves were generated by non-linear regression, and one-way ANOVA was performed with Dunnett’s multiple comparison test.

Graphs were created with GraphPad Prism 7, tables with Microsoft Office 2016, gel images and electropherograms with QIAxcel ScreenGel 1.6, and gene/primer overview with CorelDRAW X6.

All graphics have been assembled in CorelDRAW X6.

## Results

### Recovery efficiency of CMV DNA extraction from limited volumes of whole-blood from DBSs varies with sample lysis method

**Fig 2:**
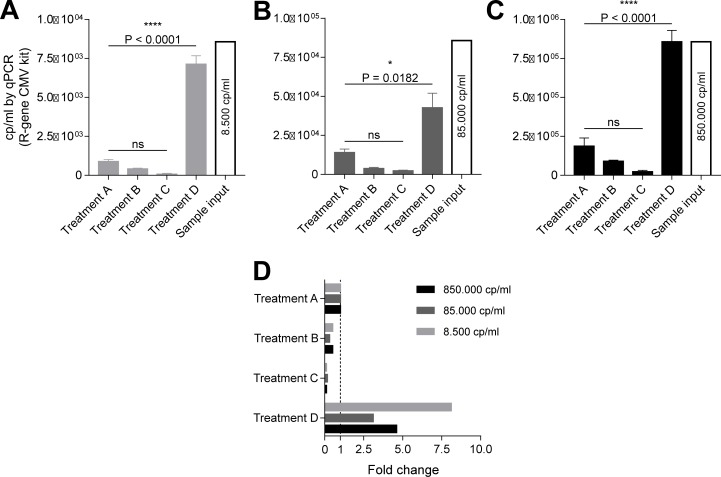
Extraction of CMV DNA from dried blood spots using four different methods. (A-C) Extraction of CMV DNA from two 3.2 mm diameter filter paper discs punched from dried blood spots with different CMV concentrations. (D) Relative extraction yield of the different treatments compared to the reference treatment (treatment A). P values were determined by one-way ANOVA; ns = not significant. n = 3.

Extracting viral DNA from DBSs for subsequent PCR analysis can be troublesome as the sample volume is often limited and the paper will retain some of the sample. Thus, we investigated different methods for the extraction of CMV DNA from DBS containing dried whole-blood spiked with a known number of CMV copies. To accommodate potential variations in blood concentration over the area of the filter paper, we punched ten 3.2 mm in diameter discs from each DBS. These discs were then randomly selected for either extraction method A, B, C, or D to prevent any selection bias for specific extraction methods. After extraction, the R-gene CMV kit was applied to determine the CMV DNA concentrations in the eluates, which was compared to the concentrations of the original samples (Fig 2 and S3 Table). We observed a large difference in recovery efficiency of CMV DNA between the different methods with extraction D being far superior to the other three (A, B, and C) at all concentrations. Also, upon visual inspection, method D appeared to remove the dried blood from the filter paper more effectively than the other methods. The mean recovery efficiency of method D ranged from 50% to 100%, extracting 19 to 113-fold more than method C, 10 to 18-fold more than method B, and 3 to 8-fold more CMV DNA than the manufacturer reference protocol (method A).

### Amplification of CMV genes UL40, UL111A, UL146, and US28 using optimized primer sets and PCR conditions is highly sensitive

The primers and PCR conditions were optimized for all four target genes (UL40, UL111A, UL146, and US28; see ‘Materials and methods*‘* for details). The placement of all evaluated primers can be seen in Fig 1 and the sequences of the best performing sets in Table 1. Sequences for all primers tested can be found in S2 Table.

**Fig 3:**
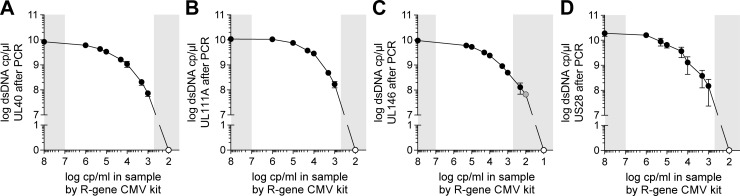
Amplicon yield of specific PCRs on diluted amniotic fluid samples with varying CMV concentrations. Successful amplifications are marked with black dots (●) and unsuccessful amplifications with white dots (○). Amplification of UL146 at 100 cp/ml was positive in two of three assays and has been marked with a grey dot (●). Grey zones mark areas above and below the limit of quantification of the R-gene CMV kit. n = 3.

After optimizing the PCR conditions for each target gene, we performed PCR on different dilutions of an amniotic fluid sample with a very high CMV concentration (approximately 100,000,000 cp/ml). As the sample had a concentration above the upper limit of quantification of the R-gene CMV kit, we first confirmed the virus concentration in several dilutions within the quantification range, which were not found to vary significantly from what was expected from the original sample. Next, PCR amplification of UL40, UL111A, UL146, and US28 was carried out using the best performing primers and PCR conditions (Fig 3).

The sensitivities of the PCRs for UL40, UL111A, and US28 were found to be close to the lower limit of detection of the R-gene CMV kit while the UL146 PCR was found to match this limit. We did not observe any non-specific amplification for any of the reactions or sample concentrations. Thus, the sensitivity of each specific PCR matched that of a commonly used diagnostic kit on dilutions of CMV positive amniotic fluid.

### Amplification of target genes from circulating wild type CMV strains in urine, serum, saliva, and bronchoalveolar lavage fluid (BALF) at low to high virus concentrations

Since the optimization and sensitivity testing of the specific PCRs were all carried out on dilutions of the same amniotic fluid-derived CMV strain, we tested whether the PCR setups were able amplify from other wild type strains in various sample types (Table 2), as both inter-strain sequence variations and PCR inhibitors inherent to the sample type could affect their performance. We included CMV positive saliva, serum, urine, and BALF with virus titers ranging from 500 to 169,000,000 cp/ml as determined by the R-gene CMV kit. The specific PCRs were able to amplify the target genes in 22 out of 23 samples. The only failed amplification was of a 500 cp/ml urine sample, which was below the concentration at which all optimized PCR reactions were positive. We did not observe any significant non-specific amplification, except for the BALF samples which was expected due to their complex nature. Together, these results suggest that our PCRs for highly variable genetic regions are sensitive to different CMV strains and work well in different types of clinical samples.

**Table 2:** Test of specific PCRs for detection of CMV target genes (UL40, UL111A, UL146, and US28) from different virus isolates in different clinical sample types (saliva, serum, urine, and bronchoalveolar lavage fluid/BALF).

Sample type	cp/ml (R-gene CMV kit)	UL40	UL111A	UL146	US28
**Saliva**	17,000	+	+	+	+
**Serum**	6,000	+	+	+	+
	11,000	+	+	+	+
	60,000	+	+	+	+
**Urine**	500	-	-	-	-
	1,500	+	+	+	+
	3,500	+	+	+	+
	4,000	+	+	+	+
	11,000	+	+	+	+
	26,000	+	+	+	+
	43,000	+	+	+	+
	43,000	+	+	+	+
	46,000	+	+	+	+
	73,000	+	+	+	+
	300,000	+	+	+	+
	700,000	+	+	+	+
	780,000	+	+	+	+
	810,000	+	+	+	+
	2,700,000	+	+	+	+
	169,000,000	+	+	+	+
**BALF**	5,000	+	+	+	+
	56,000	+	+	+	+
	90,000	+	+	+	+

Positive reactions symbolized as (+) and negative reactions as (-). Reported copy numbers have been rounded.

### Amplification of CMV genes UL40, UL111A, UL146, and US28 in DNA extracted from dried blood spots is sensitive and specific

**Fig 4:**
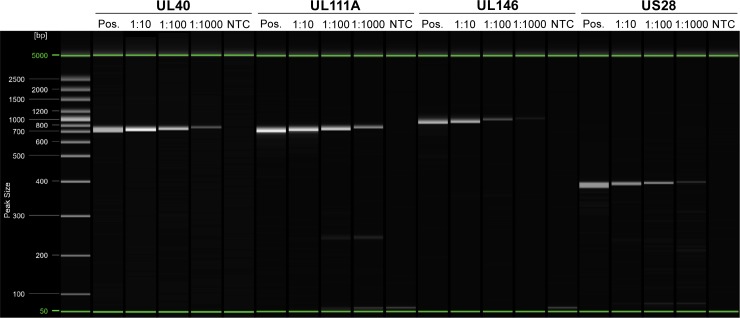
Specific PCRs for UL40, UL111A, UL146, and US28 on DNA extracted from DBSs. PCR amplification from positive sample (amniotic fluid, 100,000,000 cp/ml, Pos.), treatment D extractions from filter paper discs (1:10, 1:100, and 1:1,000 dilution; 8,500–850,000 cp/ml), and no template control (NTC). Gel representation of capillary electrophoresis reads (S1 File). Green lines represent the alignment marker used for calibration of band sizes.

Using the DNA samples obtained from DBSs by extraction method D (Fig 2), we performed PCR amplification of UL40, UL111A, UL146 and US28 using the specific primers and reaction conditions previously described in Table 1 (Fig 4). As a positive control, the high concentration amniotic fluid sample was used, which was positive in all PCRs. All the samples extracted from the DBSs were positive with the lowest CMV concentration tested at 8,500 cp/ml and highest at 850,000 cp/ml. Furthermore, we did not observe any significant non-specific amplification, but we did observe a tendency for more non-specific reads/noise at lower CMV concentrations for UL111A and US28 (S1 File). Low concentration samples were Sanger-sequenced, confirming the specificity of the amplicons (GenBank accession numbers: MN075153 [US28]; MN075154 [UL111A]; MN075155 [UL146]; MN075156 [UL40]). This suggests that DBSs can be used for detecting and genotyping CMV with high specificity and sensitivity at CMV concentrations ranging from thousands to millions.

## Discussion

A prerequisite for amplifying and genotyping CMV from stored DBSs is a robust DNA extraction method with high recovery efficiency as the available sample volume is very limited. The extraction method D reported here fulfills these criteria as up to 100% of the contained CMV DNA was recovered across different concentrations. In contrast, methods B and C had poor recovery rates at around 1%, while method A’s recovery rate (NucliSens easyMAG, bioMérieux, standard protocol) was somewhat better around 10%. Method B is a simple, cheap and fast, and thus commonly used, protocol to extract human DNA from DBSs as it does not require any extraction systems or lysis buffers. However, it comes with a few intrinsic shortfalls—the low recovery efficiency (suitable for plentiful human DNA but not for low concentrations of viruses or other microorganisms), and degeneration of DNA over time plus the presence of PCR inhibitors in the extracted sample due to the lack of DNase and PCR inhibitor removal from the whole-blood. Together with the large differences in recovery rates, there is a potential risk of not being able to subsequently detect the presence of CMV at low concentrations. Thus, it can be concluded that validation of extraction methods is an important step in order to attain optimal DNA recovery from a limited sample volume for CMV detection. A common way to circumvent this problem is the addition of a multiple displacement amplification (MDA) step prior to PCR. While MDA can amplify the amount of DNA unbiasedly by 1000 fold, many common kits requires a significant sample dilution thereby yielding little to no increase in DNA concentration. For many downstream applications, this low concentration would thus require an additional cleanup and concentrating step. Furthermore, for applications with interest in pathogen DNA, there is a significant co-amplification of human DNA which could potentially affect the sensitivity of the PCR. Since the protocol reported here (D) circumvents MDA and these additional steps, it is much faster and cheaper when implemented as part of a routine analysis.

Most studies using stored DBSs for diagnosing congenital CMV infection have not reported recovery efficiencies for their extraction protocols [15–21, 23, 24, 26–29, 44]. Exceptions are two studies using the QIAamp DNA Micro kit, reporting recovery efficiencies of 80% and 90% compared to 5% for extraction by thermal shock [22, 25]. Our data and these reports indicate that the composition of the lysis buffer is paramount for high extraction rates. However, pinpointing the critical components is unfortunately left to speculation as the composition of commercially available reagents is often regarded as a trade secret. From our results, it can be speculated that the addition of proteinase K aids the extraction of CMV DNA. This is supported by the high recovery rates also obtained in the studies using the QIAamp DNA Micro kit [22, 25], as this kit also contains Proteinase K.

High extraction rates are of no benefit if the PCR is not sensitive. Table 3 shows that the sensitivities of the PCRs for the four CMV target genes in this study are comparable to those reported by other groups, including the commercial quantitative R-gene CMV kit (also used in this study) and RealStar CMV kit [28]. This was surprising as we had designed our PCRs for genotyping of highly variable areas of the CMV genome, while the commercial kits focus purely the on detection and quantification of highly conserved CMV genes (normally UL83). Furthermore, our approach of using *Pfu*-based DNA polymerases incorporating proofreading capabilities for high-fidelity amplification, compared to the fast but error-prone *Taq* polymerase commonly used in diagnostic kits, adds another facet as the PCR products can be used for qualitative analyses, i.e. genotyping. This was confirmed by Sanger sequencing of several reactions (GenBank accession numbers: MN075153 [US28]; MN075154 [UL111A]; MN075155 [UL146]; MN075156 [UL40]), where we did not observe any problems with acquiring high quality sequences from PCR products with low DNA concentrations.

**Table 3:** Sensitivities for detection of different CMV genes by PCR as previously reported and compared to this study.

Reference	CMV gene	PCR type	Sensitivity*	
			*cp/rxn*	*cp/ml*
*1996 Clin Diagn Virol, Barbi et al. **[16]***	IE1 (= UL123)	nested PCR	40^1^ **[45]**	NR
	gp58 (= gB = UL55)		10^1^ **[46]**	NR
*2006 J Mol Diagn, Scanga et al. **[25]***	POL (= UL54)	multiplex real-time PCR	8	1600
*2007 J Clin Microbiol, Vauloup-Fellous et al. **[27]***	UL123	multiplex real-time PCR	10 **[47]**	250 **[47]**
*2008 J Clin Microbiol, Soetens et al. **[26]***	US8	PCR	NR	2000
	gH (= UL75)	nested PCR		
	UL83	real-time PCR	NR	9400
*2008 Pediatr Infect Dis J, Inoue et al. **[22]***	UL83	real-time PCR	5^1^	NR
*2010 JAMA, Boppana et al. **[19]***	gB (= UL55)	real-time PCR	NR	250
	gB (= UL55) IE2 (= UL122)		NR	50^2^
*2018 Pediatr Infect Dis J, Vives-Oños et al. **[28]***	not reported by RealStar kit	real-time PCR	NR	700 IU/ml^3^
*2019*, *Berg et al*.	UL83	real-time PCR	15	500^3^
	UL40	PCR	15	1000
	UL111A			
	UL146		3	200
	US28		15	1000

NR = Not reported in study. Rxn = Reaction.

*The sensitivities of the two commercial kits (R-gene and RealStar) as well as reference 27 is reported as LLOD, whereas as all other references report the lowest consistently positive CMV concentration.

^1^Based on detection of plasmid DNA.

^2^Based on a standard curve with lowest point of 1,200 cp/ml.

^3^According to manufacturer.

In conclusion, we here report a method for extraction of DNA from DBSs for robust detection of CMV DNA by PCR in sample volumes as low as 3–6 μl without requiring additional amplification and cleanup steps. Furthermore, we report an amplification protocol for highly variable CMV genes using a *Pfu* polymerase with proofreading capabilities and conventional PCR without compromising the sensitivity of the analysis compared to the conserved genes and *Taq*-based polymerases used in the standard diagnostic qPCR kits. Thus, as shown, the CMV DNA extracted from these samples can subsequently be used for not only quantitative but also qualitative analyses, such as genotyping of highly variable immunomodulatory CMV genes with prospects for future diagnostic purposes and risk management of CMV patients.

## Supporting information

S1 File Electropherograms of specific PCRs for UL40, UL111A, UL146, and US28 on DNA extracted from dried blood spots. Capillary electrophoresis of PCR products from positive sample (amniotic fluid, 100,000,000 cp/ml, pos. ctrl.), treatment D extractions from filter paper discs (1:10, 1:100, and 1:1,000 dilution, 8,500–850,000 cp/ml), and negative control.
(PDF)

S1 Table Historical overview of different methods used for extraction of CMV DNA from DBSs. (PDF)

S2 Table Primer sequences of all tested primers. (PDF)

S3 Table Extraction of CMV DNA from dried blood spots using four different treatments. Extraction of CMV DNA from one or two 3.2 mm diameter filter paper discs punched from dried blood spots with different CMV titers. Estimated input concentrations of CMV for one disc– 1:10 dilution: 425,000 cp/ml. 1:100 dilution: 42,500 cp/ml. 1:1000 dilution: 4,250 cp/ml. Estimated input concentrations of CMV for two discs– 1:10 dilution: 850,000 cp/ml. 1:100 dilution: 85,000 cp/ml. 1:1000 dilution: 8,500 cp/ml. Fold change in extraction yield have been calculated with treatment A as reference. Reported numbers have been rounded.
(PDF)

## Abbreviations

BALF: bronchoalveolar lavage fluid
CMV: cytomegalovirus
DBS: dried blood spot
MDA: multiple displacement amplification
PCR: polymerase chain reaction
UL: unique long region
US: unique short region
