# Supplementary figures and images for: Development of highly efficient protocols for extraction and amplification of cytomegalovirus DNA from dried blood spots for detection and genotyping of polymorphic immunomodulatory genes
Source: PLoS One. 2019 Sep 12;14(9):e0222053. doi: 10.1371/journal.pone.0222053 (PMC6742235; doi:10.1371/journal.pone.0222053)

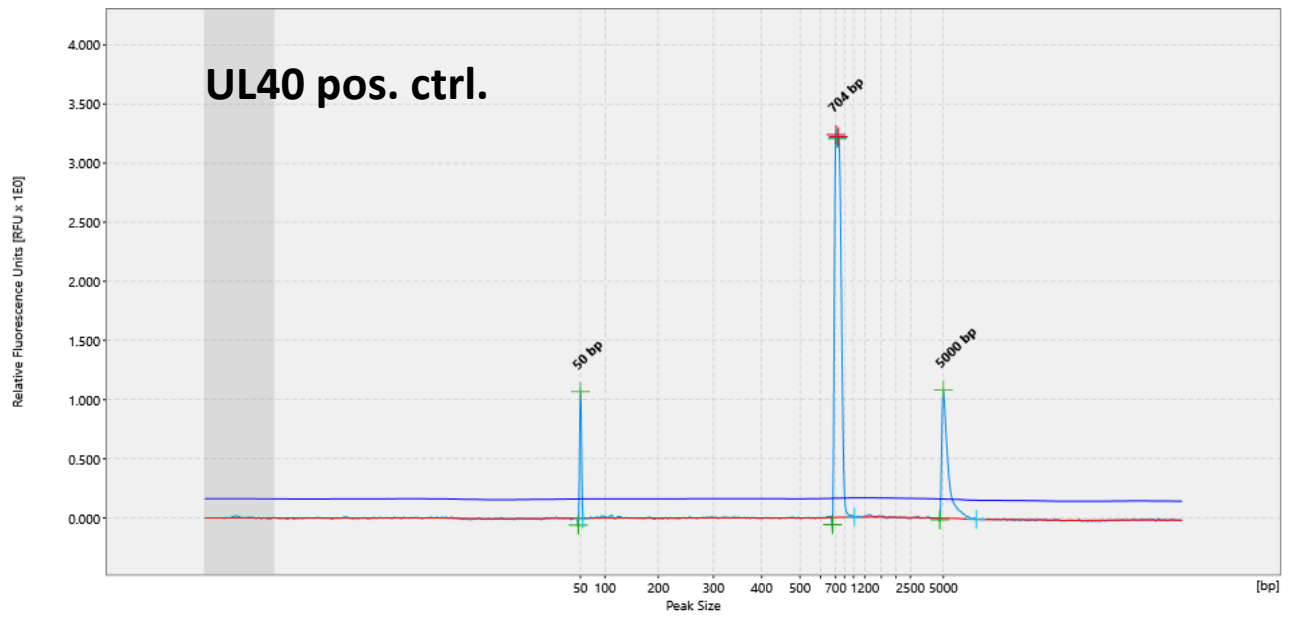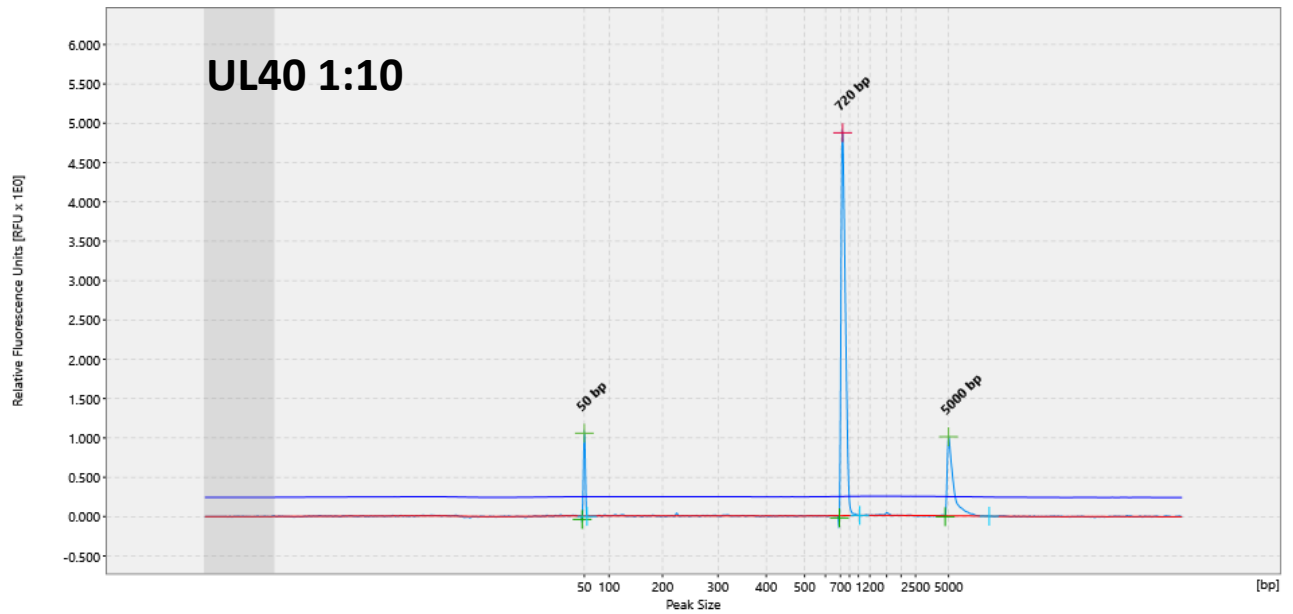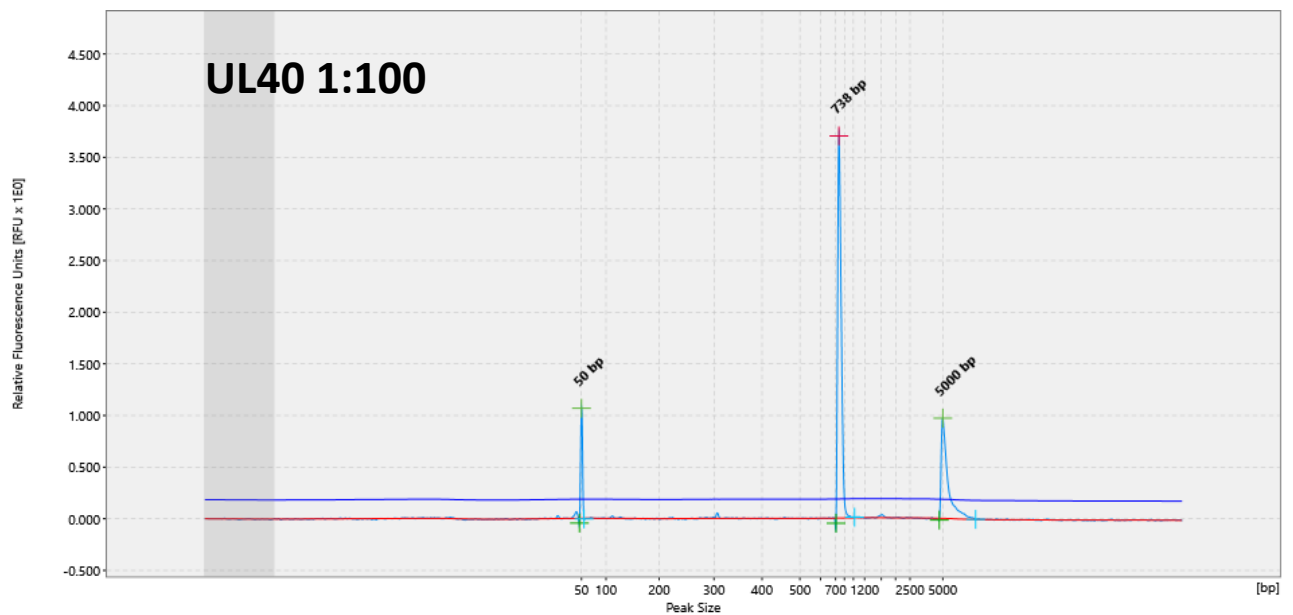

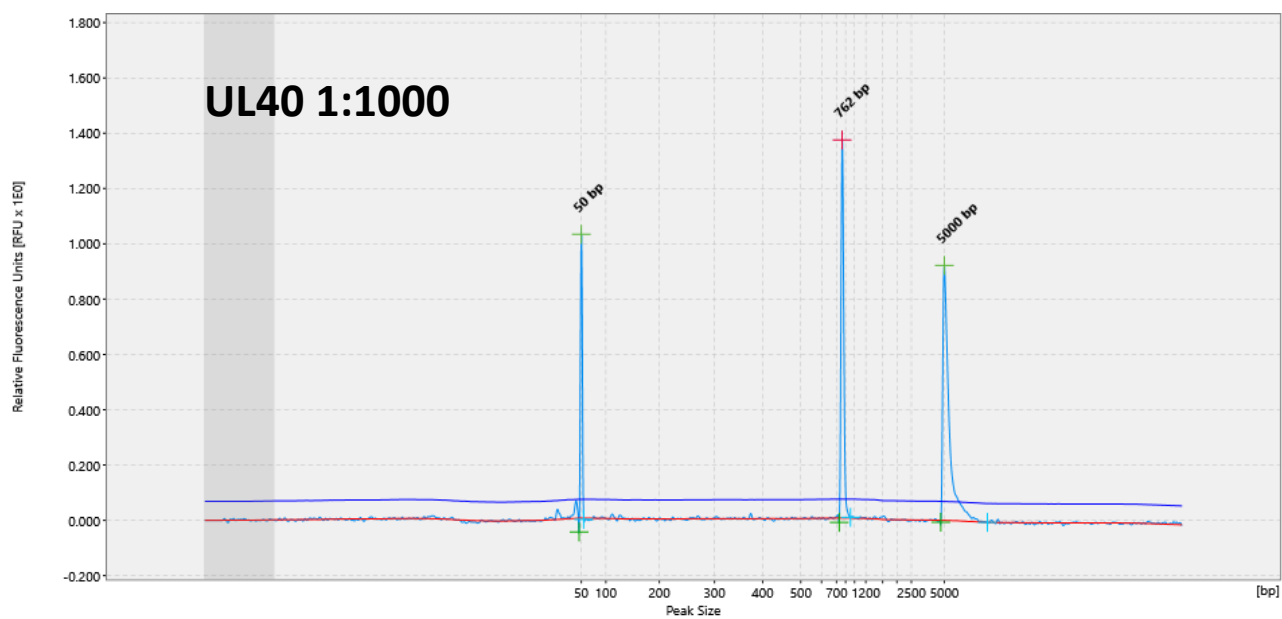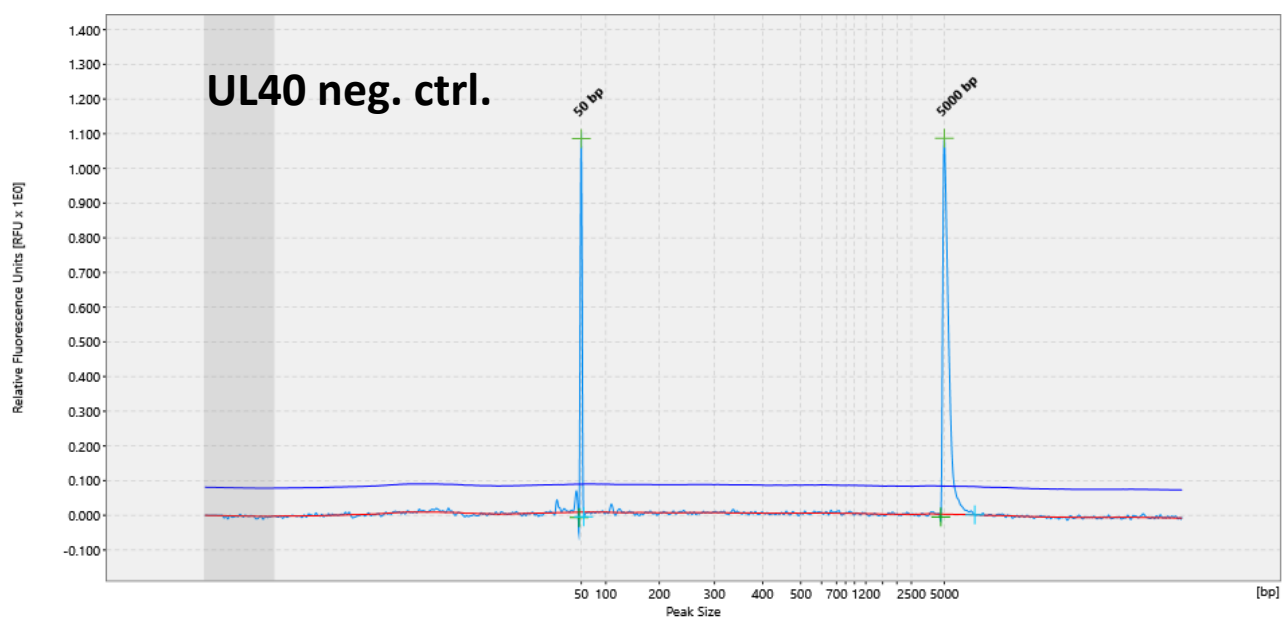

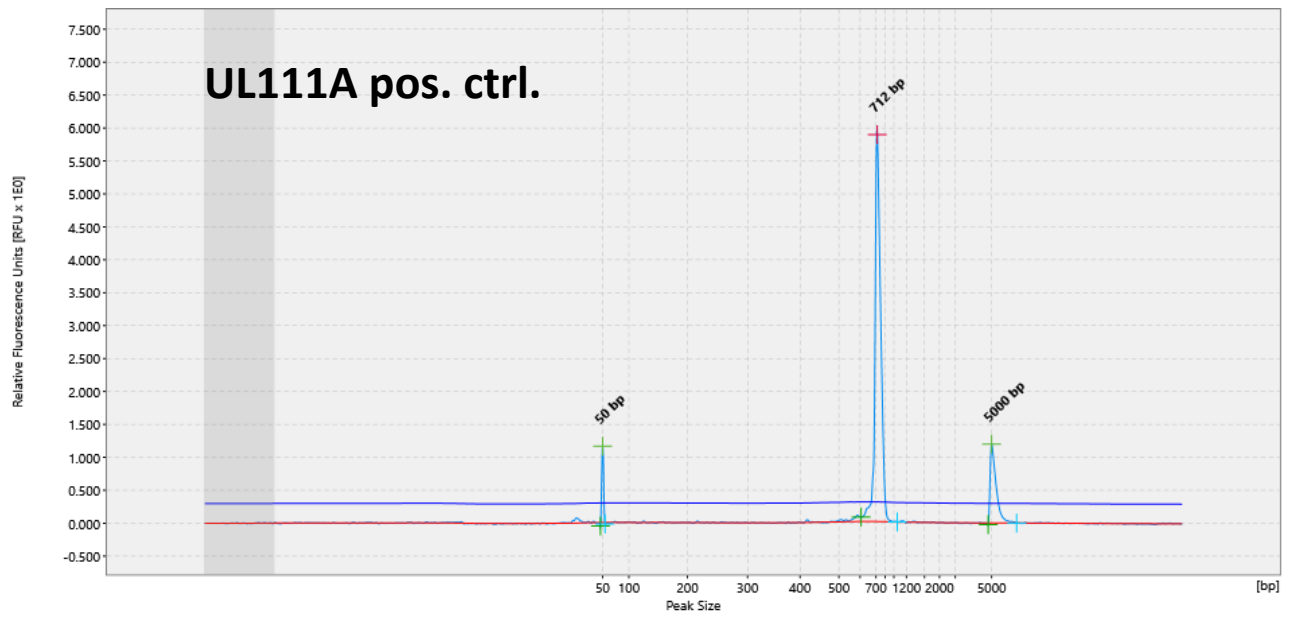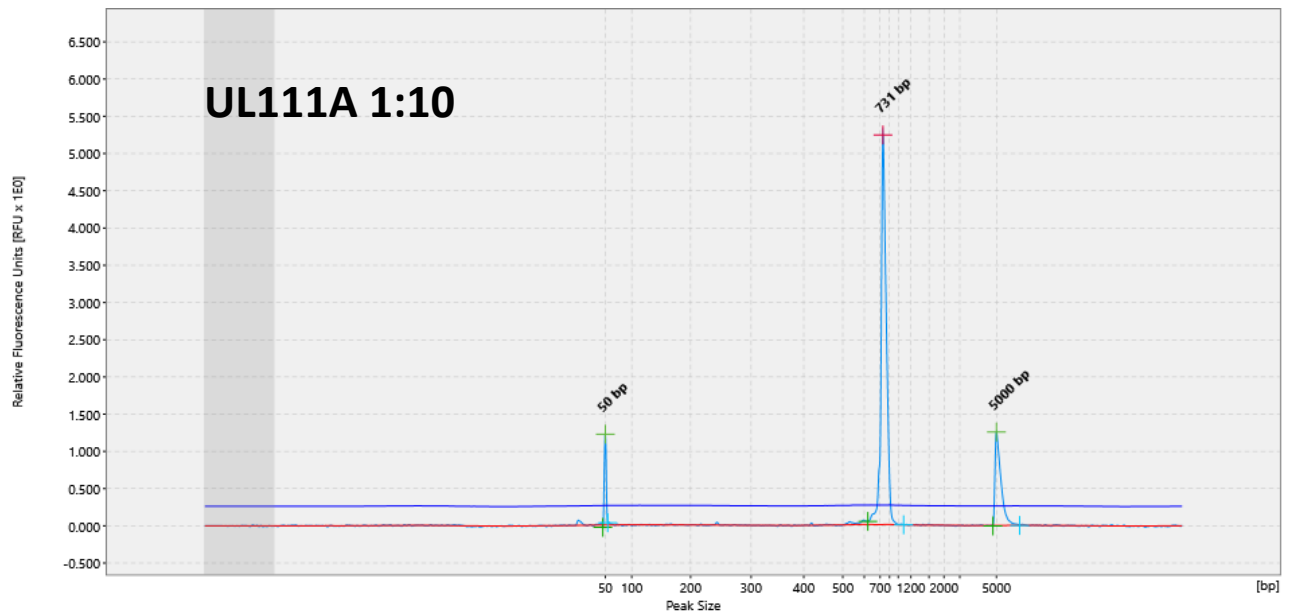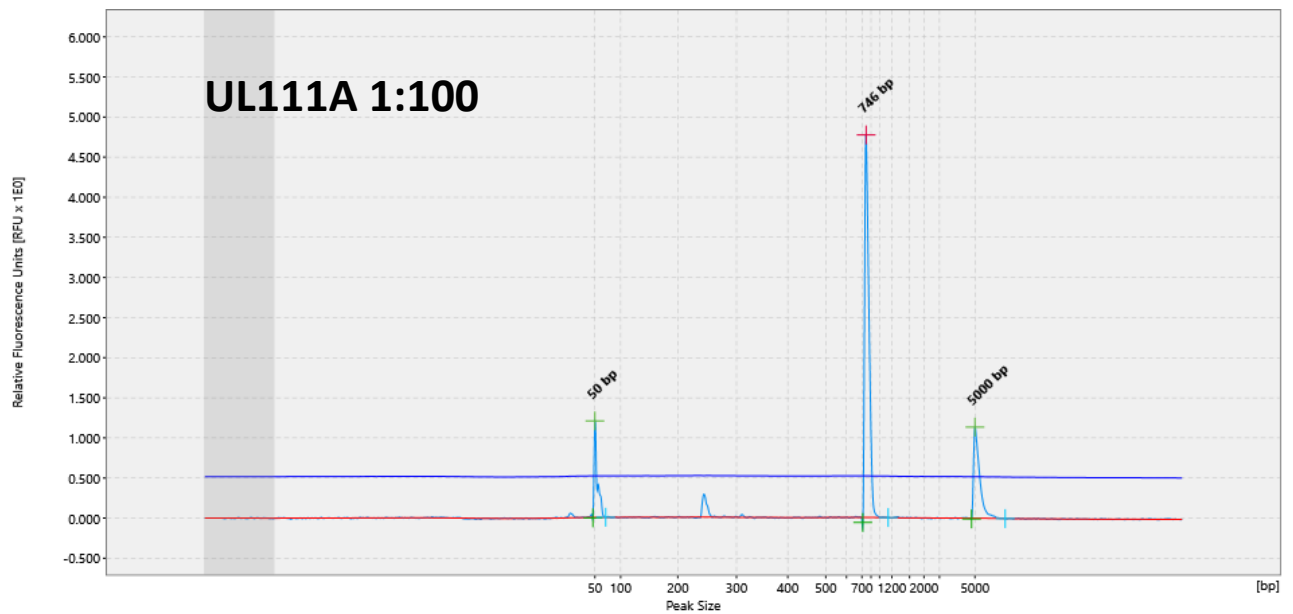

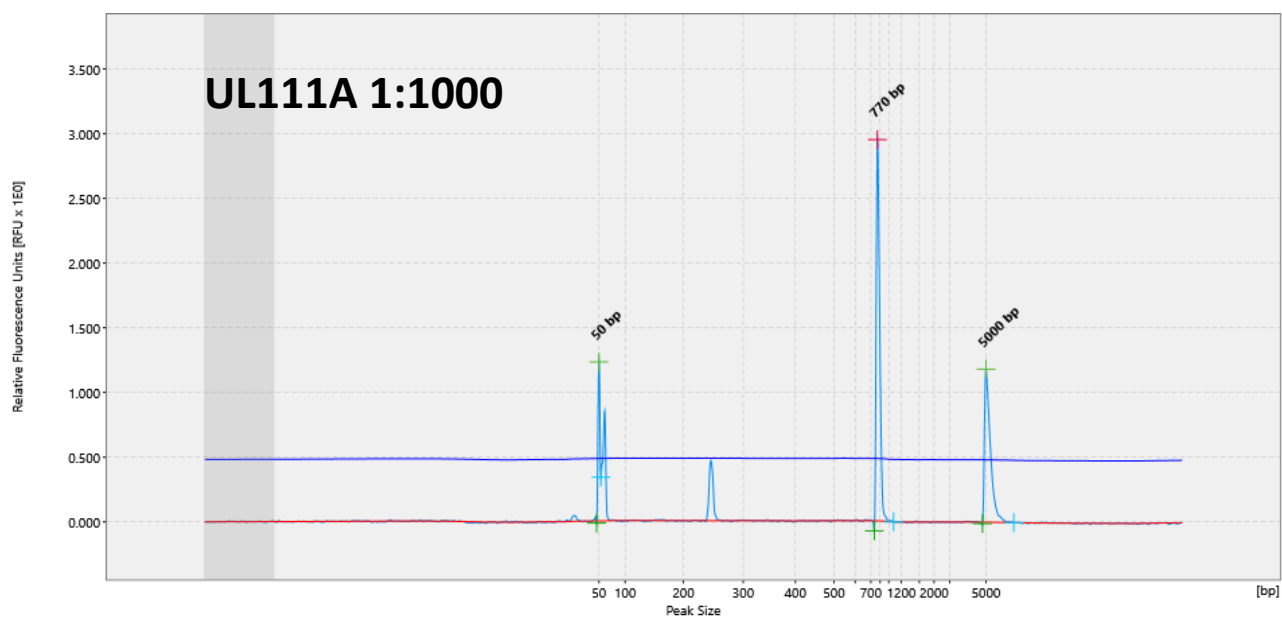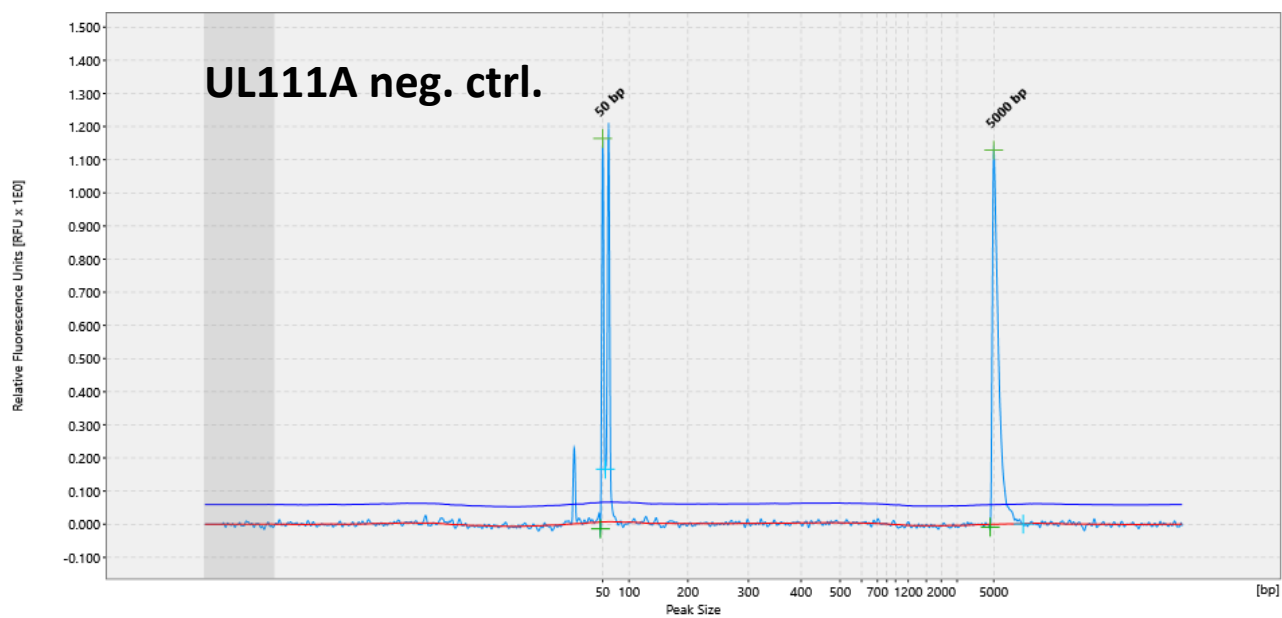

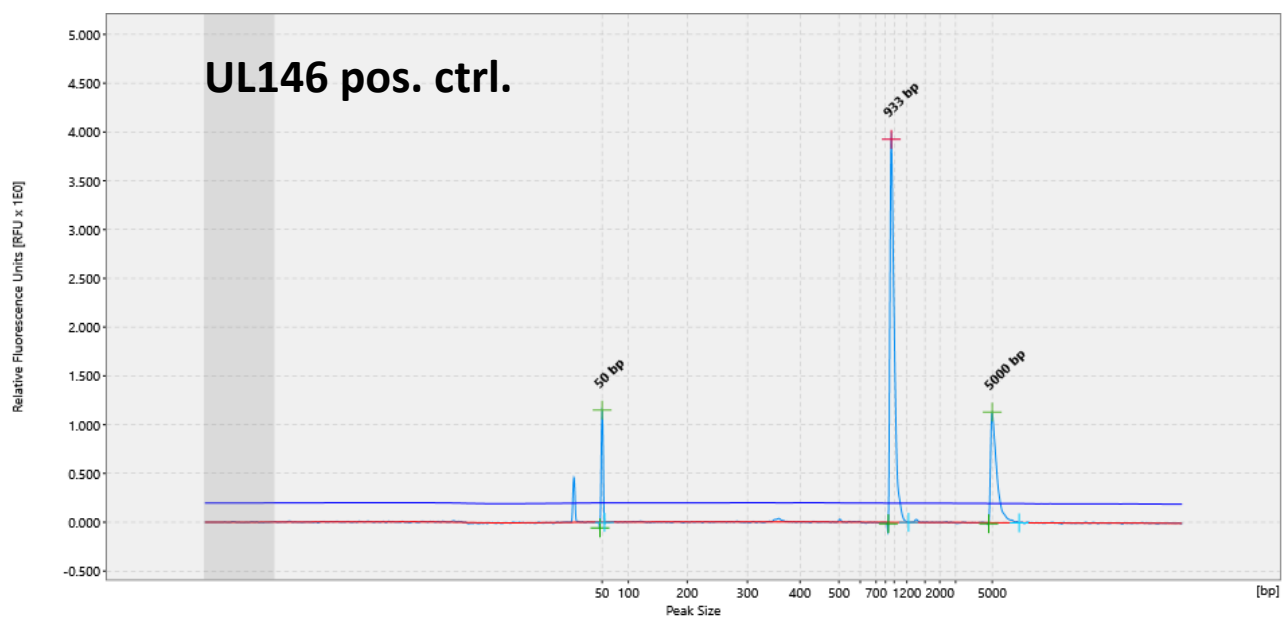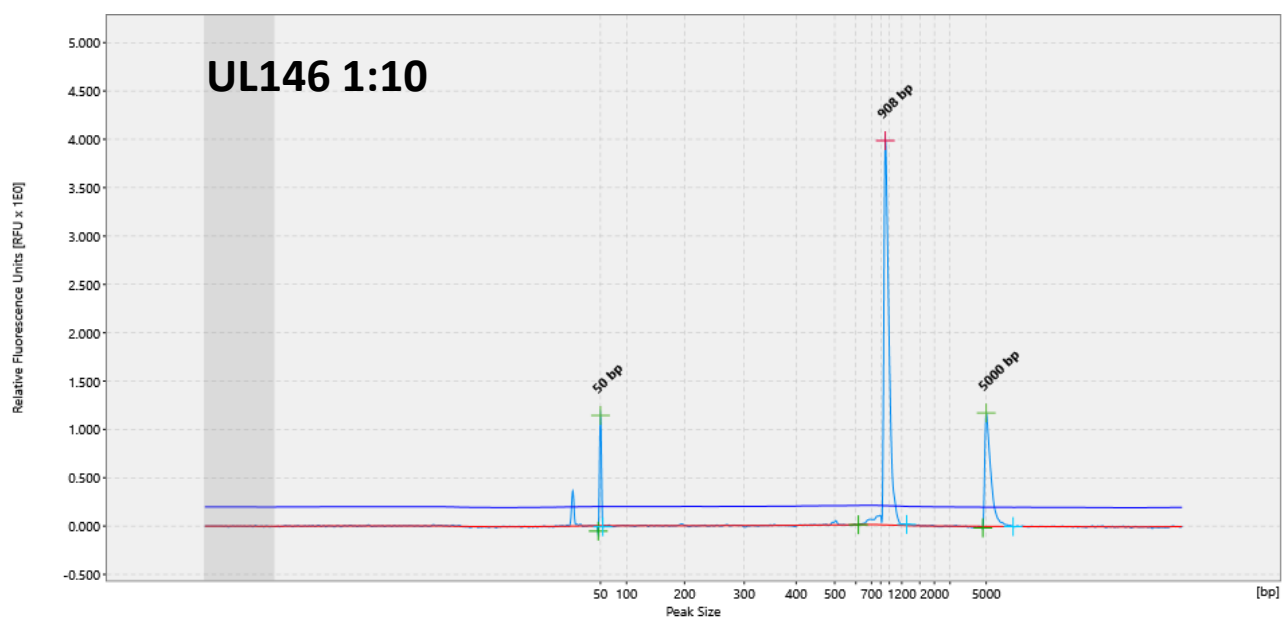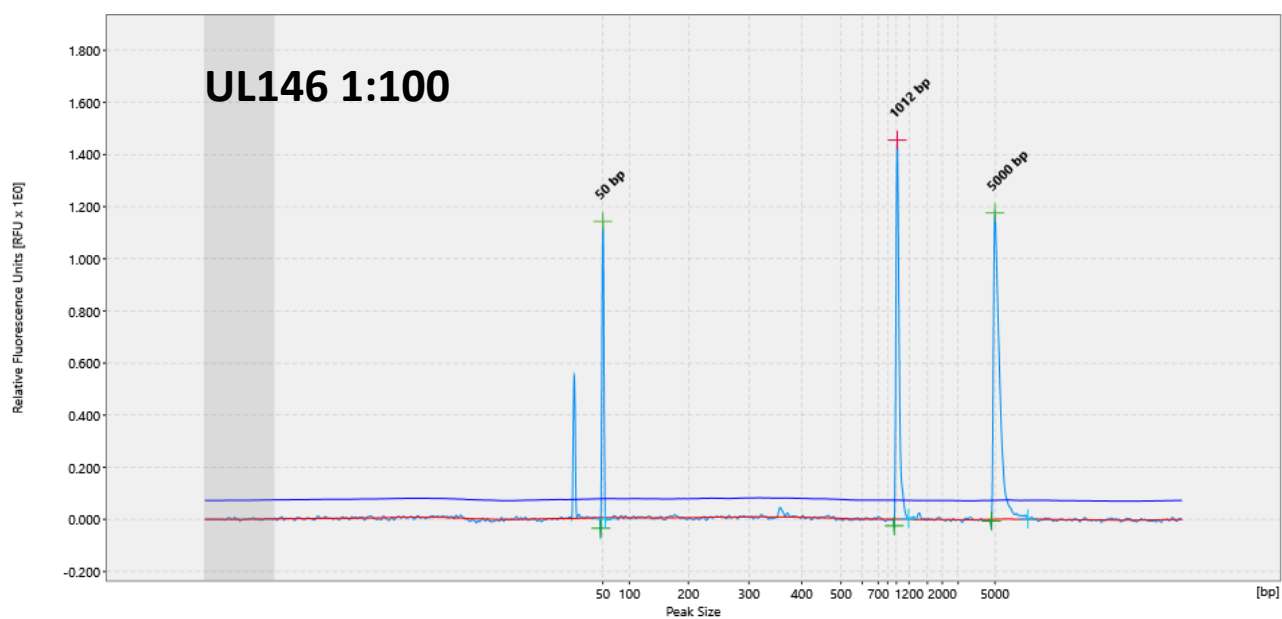

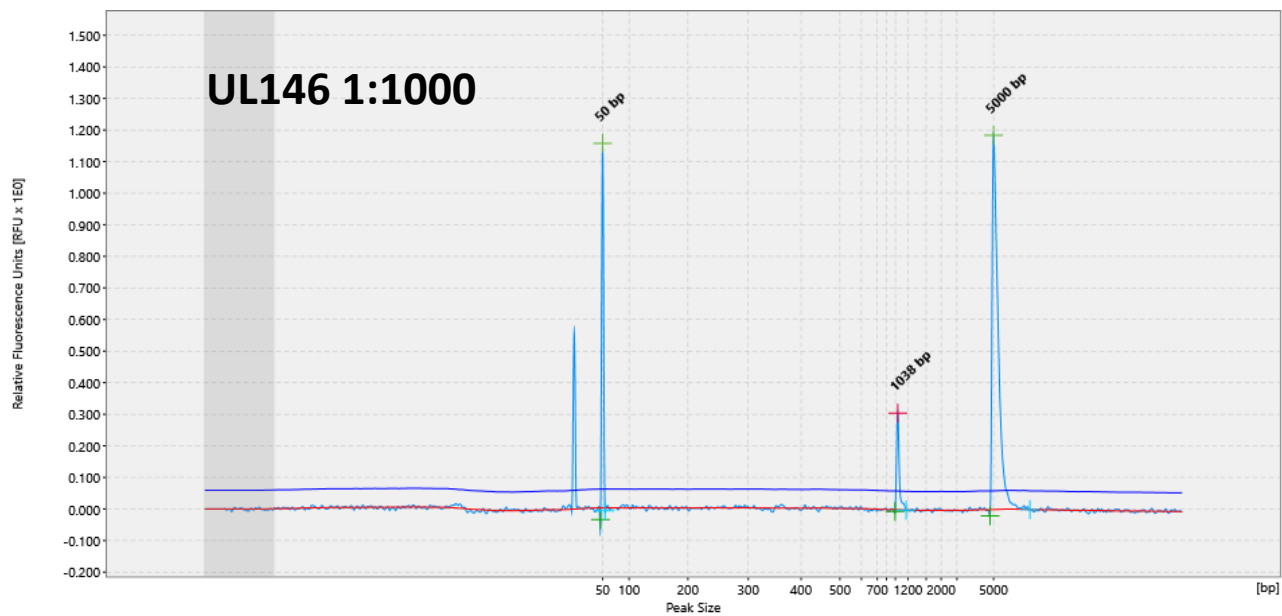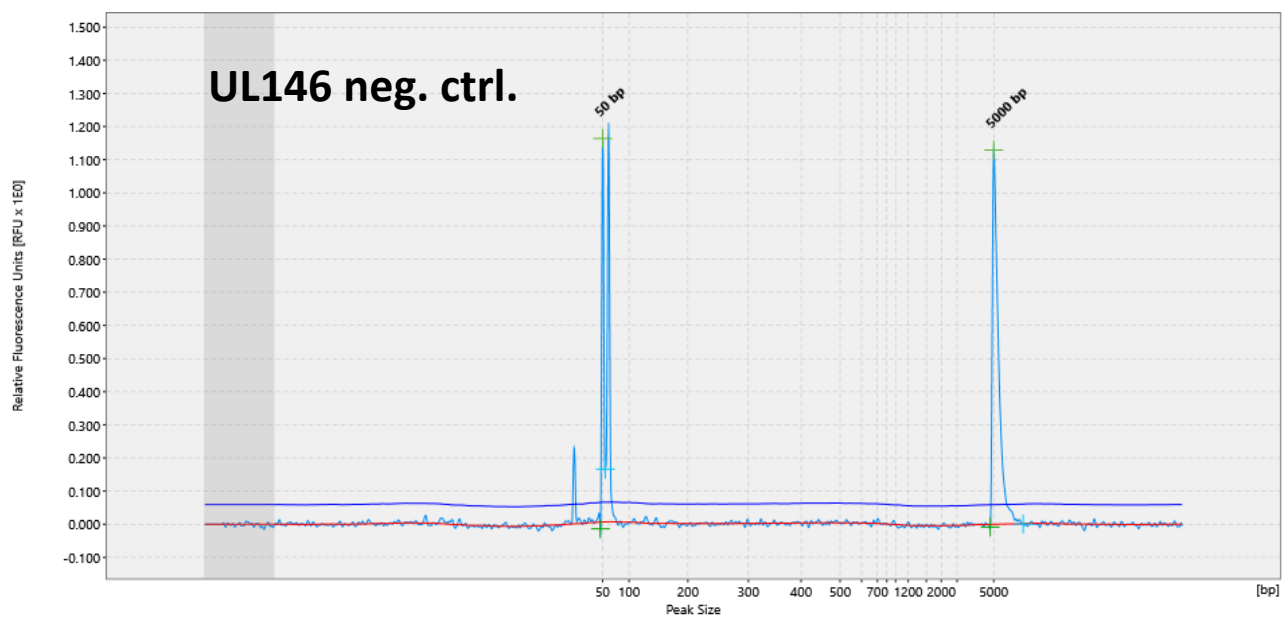

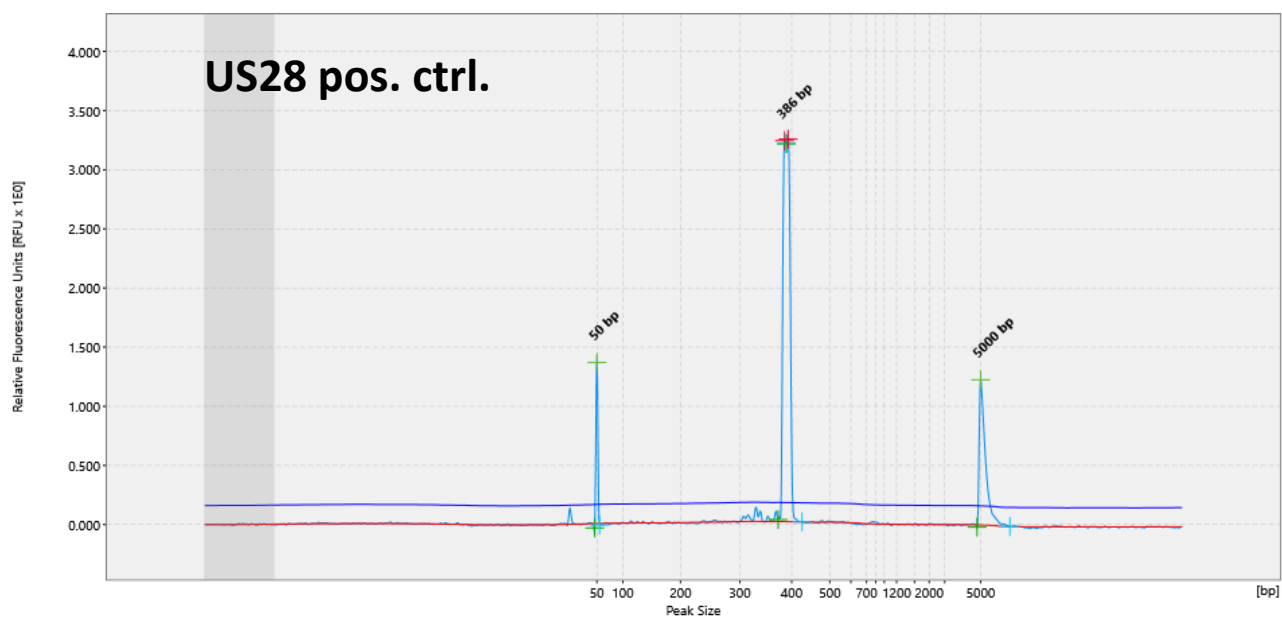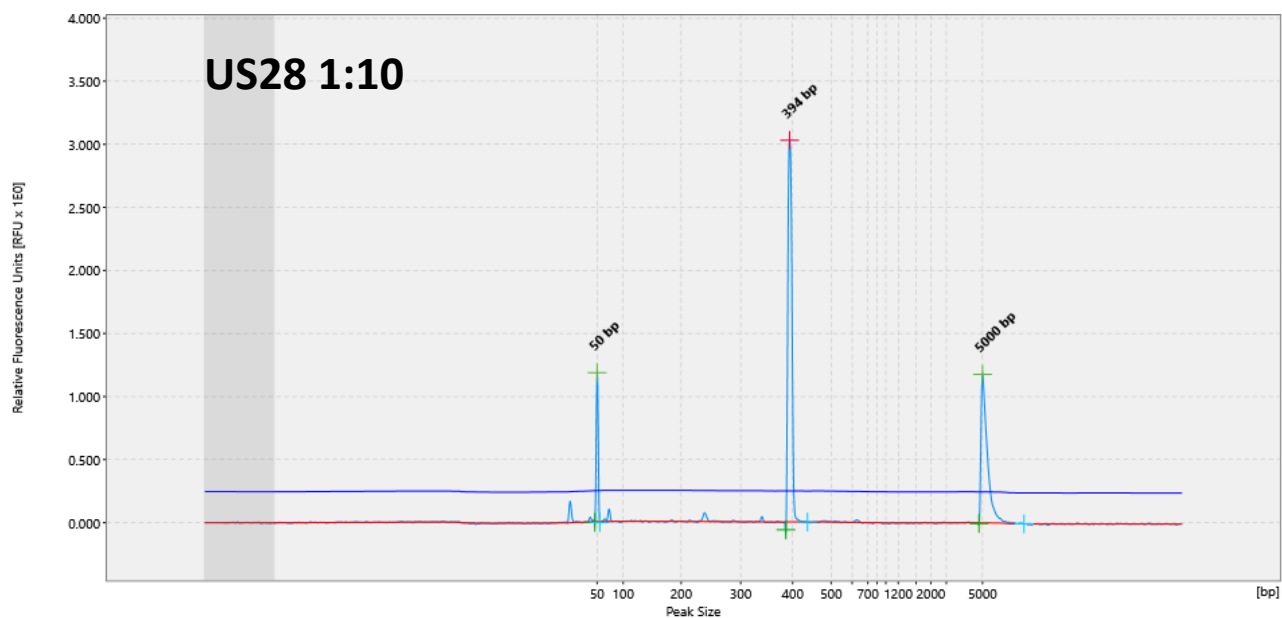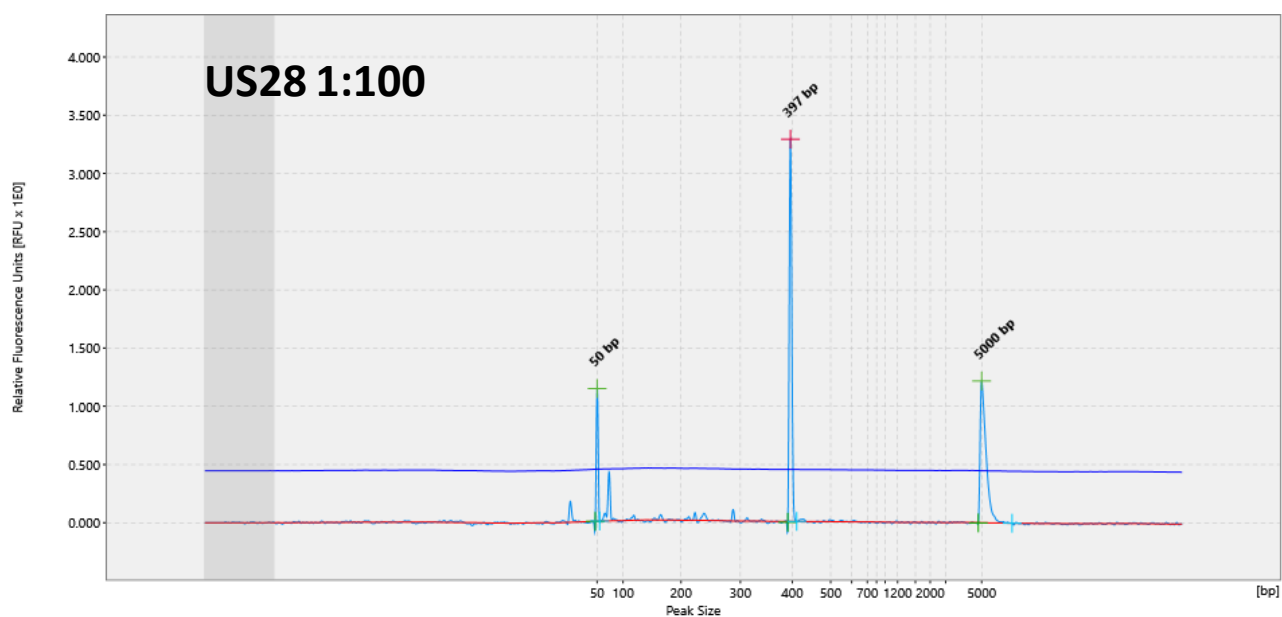

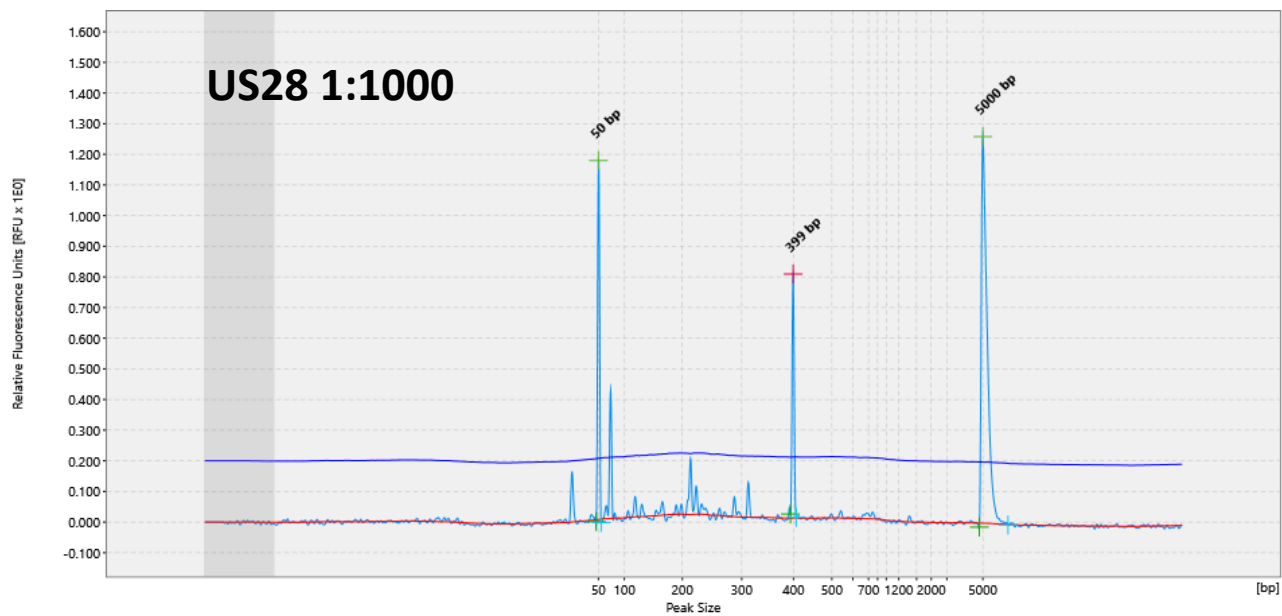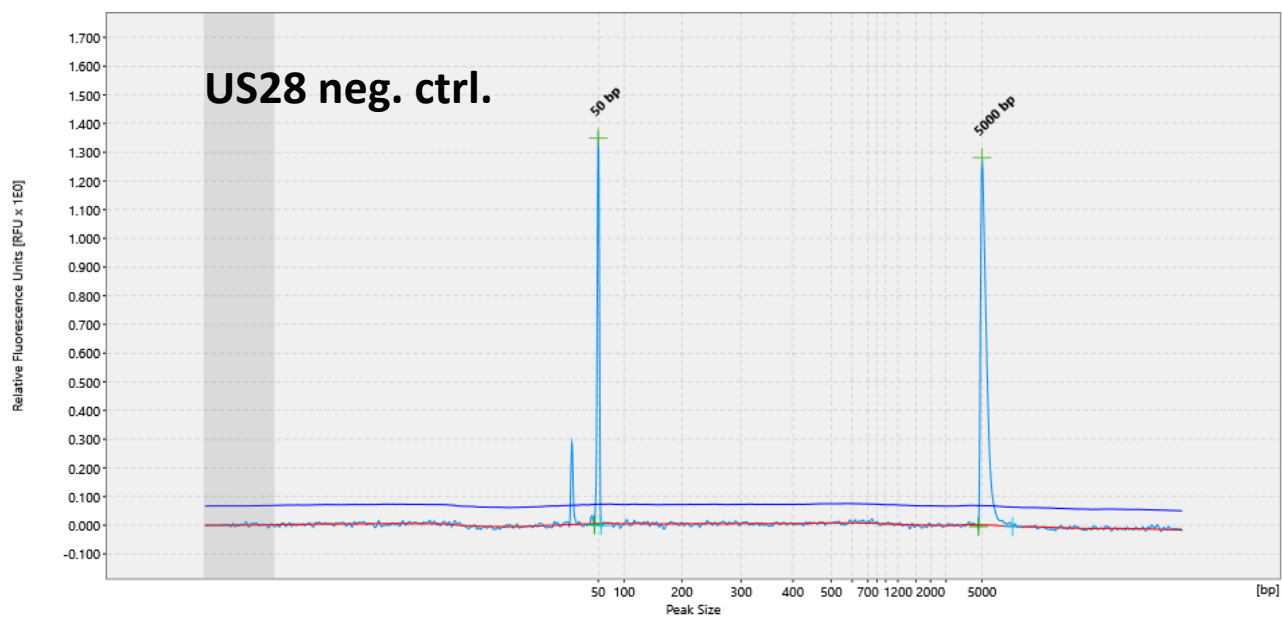

Supplement: S1 File — Capillary electrophoresis of PCR products from positive sample (amniotic fluid, 100,000,000 cp/ml, pos. ctrl.), treatment D extractions from filter paper discs (1:10, 1:100, and 1:1,000 dilution, 8,500–850,000 cp/ml), and negative control. (PDF) [file pone.0222053.s001.pdf]
