# Supplementary material for: Development of highly efficient protocols for extraction and amplification of cytomegalovirus DNA from dried blood spots for detection and genotyping of polymorphic immunomodulatory genes
Source: PLoS One. 2019 Sep 12;14(9):e0222053. doi: 10.1371/journal.pone.0222053 (PMC6742235; doi:10.1371/journal.pone.0222053)
Supplement: S1 Table — (PDF) [file pone.0222053.s002.pdf]

Reference	Extraction protocol(s)
**1996 Clin Diagn Virol, Barbi *et al.***	In water: heating to 55°C for 1 hr and 100°C for 7 min + freezing at -80°C for 30 min<br>In MEM: Heating to 55°C for 1 hr and 100°C for 7 min + freezing at -80°C for 30 min
**1997 Scand J Infect Dis, Johansson *et al.***	Lysis buffer with proteinase K (2-4 hrs at 55°C) + multiple phenol-chloroform extraction steps
**1999 Arch Dis Child Fetal Neonatal Ed, Fischler *et al.***	Lysis buffer with proteinase K (2-4 hrs at 56°C) + multiple phenol-chloroform extraction steps<br>Lysis buffer with proteinase K (overnight at 37°C) + single phenol-chloroform extraction step
**2000 J Clin Virol, Barbi *et al.***	Heating to 55°C for 1 hr and 100°C for 7 min in MEM + freezing at -80°C for 1 hr
**2001 J Virol Methods, Yamamoto *et al.***	Heating to 55°C for 1 hr and 100°C for 7 min in water
**2004 J Clin Virol, Binda *et al.***	Heating to 55°C for 1 hr and 100°C for 7 min in MEM + freezing at -80°C for 1 hr
**2006 J Clin Virol, Gohring *et al.***	Buffer with proteinase K (3-4 hrs at 56°C) + phenol-chloroform extraction<br>Heating to 55°C for 1 hr and 100°C for 7 min + freezing at -80°C for 1 hr<br>QIAmp DNA blood mini kit (Qiagen)<br>NucliSENS easyMag (Biomerieux)
**2006 J Mol Diagn, Scanga *et al.***	QIAamp DNA micro kit (Qiagen)
**2007 J Clin Microbiol, Vauloup-Fellous *et al.***	0.32% NaOh + QIAamp DNA blood mini kit (Qiagen)<br>Lysis buffer with proteinase K (for 1 hr at 56°C) + phenol-chloroform extraction
**2008 J Clin Microbiol, Soetens *et al.***	Lysis buffer with proteinase K (for 1 hr at 56°C) + phenol-chloroform extraction<br>NucliSENS easyMag (Biomerieux)
**2008 Pediatr Infect Dis J, Inoue *et al.***	Heating to 55°C for 1 hr and 100°C for 7 min in MEM + freezing at -80°C for 1 hr<br>QIAamp DNA micro kit (Qiagen)
**2009 J Clin Virol, De Vries *et al.***	Heating to 55°C for 1 hr and 100°C for 7 min in MEM + freezing at -80°C for 1 hr<br>QIAamp DNA Investigator Kit (Qiagen)<br>BioRobot Universal System (Qiagen)<br>MagNA Pure LC (Roche)<br>QIAsymphony (Qiagen)<br>NucliSENS easyMag (Biomerieux)<br>Dynabeads Silane (Invitrogen)
**2009 J Med Virol, Atkinson *et al.***	QIAmp DNA blood mini kit (Qiagen)
**2010 JAMA, Boppana *et al.***	Qiagen BioRobot M48 with MagAttract<br>Qiagen “column DNA extraction”
**2015 J Clin Virol, Koontz *et al.***	QIAamp DNA Investigator kit<br>QIAamp DNA Investigator kit with QIAcube automation<br>QIAamp DNA mini kit<br>MagAttract DNA Mini kit with M48 automation<br>Thermal Shock<br>Genta Puregene
