# Supplementary material for: Development of highly efficient protocols for extraction and amplification of cytomegalovirus DNA from dried blood spots for detection and genotyping of polymorphic immunomodulatory genes
Source: PLoS One. 2019 Sep 12;14(9):e0222053. doi: 10.1371/journal.pone.0222053 (PMC6742235; doi:10.1371/journal.pone.0222053)
Supplement: S2 Table — (PDF) [file pone.0222053.s003.pdf]

Primer target	Set number	Forward sequence (5'-3')	Reverse sequence (5'-3')
UL146	1	**CCGGGAATACCGGATATTACG**	**CAGCACTTCCTGACGATTG**
	2	GAGCTGTCATTGTAGAATTAGTC	GCGCGTTAGGATTGAGACAC
	3	TCATGCCTGTTGAGATGCTC	ATAGCCAGCACTTCCTGACG
	4	GGGAATACCGGATATTACG	AAATAGCCAGCACTTCCTG
UL111A	1	CATAAAGGACCACCTACCTGG	CACACGGAGTAGTCGTCCTC
	2	GATGGTCTCTTCCTCTCTG	CACGGACTTATCTCCGCAACC
	3	TCATAACATAAAGGACCACC	GCAACGTGGTTAAACAGTAC
	4	**CATCATAACATAAAGGACCACCTAC**	**CTGAGACAGCCGACTAATCAC**
UL40	1	CTGCCTACGTCTTCATGACG	CCTCATACACAGGAGTCC
	2	CTGTCTCGTCGTCATTCGG	CCATGTCGCCTTGGTGGTAC
	3	**CTCTGTCTCGTCGTCATTC**	TTCAAGGCGTAGTGATGATC
	4	CTGCCTACGTCTTCATGAC	CTCCTGATCCTCATACACAG
	5	CTGCCTACGTCTTCATGA	**GAATGCCCACAGTGTACATG**
	6	TCCACGTTGAAAGCTTTCTAC	TAAGGGCACTCGTGAGGATG
US28	1	GCTCGAAGAGGTTGGTGTG	CAACGCCGTACAGAAACAACG
	2	**CCGCTCATATAGACCAAACC**	**AGGGAGTTGTGATCTAGGAG**
