# Supplementary material for: Development of highly efficient protocols for extraction and amplification of cytomegalovirus DNA from dried blood spots for detection and genotyping of polymorphic immunomodulatory genes
Source: PLoS One. 2019 Sep 12;14(9):e0222053. doi: 10.1371/journal.pone.0222053 (PMC6742235; doi:10.1371/journal.pone.0222053)
Supplement: S3 Table — Extraction of CMV DNA from one or two 3.2 mm diameter filter paper discs punched from dried blood spots with different CMV titers. Estimated input concentrations of CMV for one disc– 1:10 dilution: 425,000 cp/ml. 1:100 dilution: 42,500 cp/ml. 1:1000 dilution: 4,250 cp/ml. Estimated input concentrations of CMV for two discs– 1:10 dilution: 850,000 cp/ml. 1:100 dilution: 85,000 cp/ml. 1:1000 dilution: 8,500 cp/ml. Fold change in extraction yield have been calculated with treatment A as reference. Reported numbers have been rounded. (PDF) [file pone.0222053.s004.pdf]

	1:10 dilution						1:100 dilution						1:1000 dilution					
**Treatment**	*cp/ml*	±	*SEM*	*n*	*Recovery eff.*	*Fold change*	*cp/ml*	±	*SEM*	*n*	*Recovery eff.*	*Fold change*	*cp/ml*	±	*SEM*	*n*	*Recovery eff.*	*Fold change*
1 disc																		
A	123,000	±	13,000	3	0.29	1	8,000	±	3,000	3	0.19	1	500	±	200	3	0.11	1
B	35,000	±	17,000	3	0.08	0.3	2,500	±	500	3	0.06	0.3	100	±	50	3	0.03	0.2
C	24,000	±	1,000	3	0.06	0.2	1,500	±	500	3	0.03	0.2	25	±	25	3	0.01	0.1
**D**	**432,000**	±	**144,000**	**3**	**1.01**	**3.5**	**31,000**	±	**2,000**	**3**	**0.73**	**3.8**	**3,000**	±	**100**	**3**	**0.66**	**6.0**
2 discs																		
A	187,000	±	53,000	3	0.22	1	14,000	±	2,000	3	0.16	1	1,000	±	100	3	0.10	1
B	89,000	±	8,000	3	0.10	0.5	4,000	±	1,000	3	0.04	0.3	500	±	50	3	0.05	0.5
C	22,000	±	10,000	3	0.03	0.1	2,000	±	500	3	0.02	0.2	50	±	50	3	0.01	0.1
**D**	**856,000**	±	**73,000**	**3**	**1.00**	**4.6**	**43,000**	±	**9,000**	**3**	**0.50**	**3.1**	**7,000**	±	**500**	**3**	**0.83**	**8.1**
